# Factors Influencing Consumers’ Perceptions of Food: A Study of Apple Juice Using Sensory and Visual Attention Methods

**DOI:** 10.3390/foods8110545

**Published:** 2019-11-03

**Authors:** Katarzyna Włodarska, Katarzyna Pawlak-Lemańska, Tomasz Górecki, Ewa Sikorska

**Affiliations:** 1 Institute of Quality Science, Poznań University of Economics and Business, al. Niepodległości 10, 61-875 Poznań, Polandkatarzyna.pawlak-lemanska@ue.poznan.pl (K.P.-L.); 2 Faculty of Mathematics and Computer Science, Adam Mickiewicz University, Uniwersytetu Poznańskiego 4, 61-614 Poznań, Poland; tomasz.gorecki@amu.edu.pl

**Keywords:** apple juice, consumer perception, internal preference mapping, visual attention, packaging, label

## Abstract

The aim of this study was to evaluate the influence of intrinsic product characteristics and extrinsic packaging-related factors on the food quality perception. Sensory and visual attention methods were used to study how consumers perceive the quality of commercial apple juices from four product categories: clear juices from concentrate, cloudy juices from concentrate, pasteurized cloudy juices not from concentrate, and fresh juices. Laboratory tests included the assessment of sensory liking in blind and informed conditions and expected liking based on packages only. The results showed that brand and package information have a large impact on consumers’ sensory perceptions and generate high sensory expectations. An innovative visual attention tracking technique was used in online experiments to identify packages and label areas on individual packages, which attracted consumer attention. During an online shelf test, consumers mostly focused on not from concentrate juices from local producers, which were perceived as more natural, healthy, and expensive than juices reconstituted from concentrate. When individual labels were analyzed, consumers predominantly focused on nutritional data, brand name, and information about the type of product. The present results confirm a large impact of information and visual stimuli related to packaging on product perception.

## 1. Introduction

The sources that consumers use to form their impression of a product are typically classified as intrinsic or extrinsic cues [1,2]. The intrinsic cues are those characteristics that are part of the physical product. Some of these may be assessed before consumption (e.g., color, size, damage), while others may only be experienced through consumption, i.e., sensory properties. There is no single defined set of sensory attributes that are important across all of the food products. The importance of flavor, texture, and appearance attributes is product-dependent [3]. The extrinsic sources of information are those that are related to the product, but are not a part of it physically, such as brand name, label, packaging, price, the location where it is sold, and marketing communications [3,4]. These external cues generate consumer expectations about food products and affect their choices, sensory perception, and hedonic liking [5,6]. For these reasons, both sensory and non-sensory aspects should be included into the consumer research of food quality perception.

Sensory consumer research has confirmed that extrinsic product cues affect consumer perception of the food product sensory quality [4,7,8]. It has been reported that the brand name shown on a package influences consumer liking of taste of various food products [9,10]. The nutritional labeling also affects the estimation of taste [11]. In contrast, little is known about the relative effect of the extrinsic cues on the informed product evaluation when multiple cues are present [4].

The consumer perception of food packages and labels has been traditionally based on the self-reported estimates, although these estimates are subject to different biases and are reportedly only a poor indicator of the consumer behavior in real-life situations [6]. Some recent studies demonstrated that eye-tracking methodology is adequate for examining the effect of the package features on the consumer attention [12]. Food packages and nutrition labels were investigated with eye-tracking tools to monitor consumer visual attention to the respective product and nutritional information [13,14,15]. The eye-tracking technology precisely tracks the location and duration of gaze, providing detailed information on the consumer’s visual attention and is more and more widely used in product marketing and consumer science, and recently also in consumer sensory studies [16,17].

Apple juice is one of the most popular products among juices all over the world, due to its pleasant sensory qualities and high nutritional value. Presently, the juice market offers a wide range of differently processed products. New trends in the juice market are determined by the consumer demand [18,19]. One of the fastest growing trends is that for minimally processed products, characterized by sensory properties similar to those of the fresh material and preserving valuable components of the fresh fruit. Apple juices available on the market include natural juices obtained directly from fruit by squeezing, not from concentrate (NFC) juices, both pasteurized and unpasteurized, and juices reconstituted from concentrate (FC), clear varieties and pulp-enriched cloudy varieties. Within each of the groups, one can distinguish ecological and conventional products. According to the European Fruit Juice Association (AIJN) Liquid Fruit Market Report [20], the consumption of non-concentrate fruit juices has increased significantly over the last five years in the European Union. The information on the production process is mandatory on the juice package. The results of previous studies showed varied consumer preferences for apple juices from different product categories [21,22].

Commercial apple juices are available in a variety of packages, including glass or plastic bottles and cartons. Food packaging is primarily used to protect food products from environmental contamination and other influences, and to ensure food quality and safety [23]. However, the packaging also has an important communicative role. The effective communication of product advantages through the packaging design determines consumer impressions of the product quality [24]. Thus, consumers mostly prefer products that attract their visual attention; therefore, food packaging must also be attractive or eye-catching. Both shape, material, color, and layout of the packaging elements have been shown to impact consumer perception [25]. Therefore, more research is needed in order to identify the packaging features that attract attention and enhance the buying potential.

The aim of the present work was to study the influence of the intrinsic product properties and the extrinsic cues related to packaging upon the food quality perception and liking. We studied commercially available apple juices from various product categories. We investigated the influence of the intrinsic cues and packages on liking and expectations using a consumer sensory study. Consumer visual attention to the apple juice packages and labels was examined with the use of an online visual attention tracking technique.

## 2. Materials and Methods 

### 2.1. Apple Juice Samples

Eight apple juices from four product categories available on the Polish market were evaluated in this study. Two representative juices produced in Poland were selected in each of the four market categories, based on our previous studies of the physicochemical and sensory properties of a large number of juices and public recognition of their brands [26,27]. The selected products included juices reconstituted from concentrate (clear and cloudy varieties) and direct naturally cloudy juices (pasteurized and freshly squeezed varieties). Taking into account the type of production, the package, and the price, the juices studied may be classified into the standard and premium market segments. The detailed description of the samples is presented in Table 1. 

### 2.2. Consumer Studies

Two experiments were performed to evaluate consumer perceptions of the commercial apple juices using different complementary methodologies. All of the participants declared to consume apple juices at least several times a month and to have apple juice as the first preference among fruit juices. No ethical approval was required for this study. The first experiment was performed in laboratory conditions and the second experiment online. The experiments were conducted as follows:

Experiment 1. A group of 96 consumers of apple juices participated in the laboratory sensory study (64.6% female and 35.4% male). The participants were recruited from staff and students of the Poznań University of Economics and Business based on their availability and interest in participation in the study. Of this number, 55.2% were under 25 years old, 36.5% were between 26 and 45 years old, and 8.3% were over 45 years old. The same group of consumers examined the quality of apple juice samples in different exposure conditions. To prevent communications between the members of the consumer panel, they were informed about the rules of the experiment, each person occupied a separate cubicle, and the sessions were supervised.

Two sessions were held over two consecutive weeks. Three types of data were gathered with the same group of consumers: (1) overall liking of the samples in blind-testing conditions, (2) expected liking of juices in packages, and (3) overall liking of the samples in an informed testing conditions; all samples were evaluated on 9-point hedonic scales (1 = dislike extremely, 5 = neither like nor dislike, and 9 = like extremely). The samples were presented to the consumers following a balanced rotation scheme. In blind-testing experiments, juices were coded by three-digit random numbers.

Fifty milliliters of each beverage were served to consumers at 20 °C in transparent plastic containers in blind and informed sensory evaluations, respectively, without and with packages. Mineral water was available during studies. The results of this experiment were already presented in our previous paper [28], where blind liking scores were discussed in detail and the relationships between the consumer liking and the physicochemical and sensory properties of juices were determined.

In an expected liking evaluation, consumers were asked to inspect full packages of eight apple juices and to score their expected sensory liking. To evaluate spontaneous perception of the labels, without making participants focus on specific aspects, they were asked to answer check-all-that-apply (CATA) questions with terms describing the product characteristics: natural, artificial, tasty, tasteless, not very healthy, very healthy, expensive, inexpensive, a familiar product, an innovative product, and a trendy product. Consumers were asked to check all the terms they considered appropriate to describe each of the products. 

Experiment 2. A group of 171 consumers (68.8% female and 31.2% male) participated in the online study using Attensee software (https://www.attensee.com). Of these, 72.9% were under 25 years old, 23.0% were between 26 and 45 years old, and 4.1% were over 45 years old.

Attensee is an application used for the study of visual attention, that mimics an eye-tracker [29]; it provides an interface for computers and tablets and does not require any special hardware. This innovative approach to record human information lets one see what consumers look at on labels, packaging, or pictures. To get the required information, the reference areas (RA) on packaging and labels should be marked, and the percentage of consumers focusing their attention on these RA are recorded [30]. Based on these results, final calculations of the visual path tracking parameters may be made. The following RA were used in our experiments: brand names, logos, pictograms, photos, tables and lists of nutritional values, slogans regarding the content of pro-health ingredients, eco/bio labeling, and packaging size statement.

This experiment was conducted online. Consumers were asked to take part in the study via social media, and after agreeing, they received an individual link to the online experiment and were informed that the study involved the simulation of visual perception on a computer screen by means of cursor movements. The participants used the mouse pointer to bring certain parts of the screen into clarity (focused circle 2 cm in diameter). The software updated the information in real time as participants changed their area of focus and the way of exploration. After receiving instructions and doing some trial runs, participants performed the actual test. First, an image of all of the analyzed products on the shop shelf was presented on a computer screen for 45 s, and then the images of individual juice labels were presented, each for 30 s. The sequence of showing the individual labels was proportional and randomized across participants to eliminate the order effects. Each of the participants examined an image with all of the products (shelf picture), and randomly selected four of the eight individual product images. The photographic images of juice packages (with 4592 × 3056 pixels resolution) were captured using a digital camera (Sony A3056, Tokyo, Japan). 

The Attensee software can also produce different types of questionnaires. Thus, the participants were asked to answer a check-all-that-apply questionnaire with the terms describing the product, the same as in Experiment 1. 

### 2.3. Data Analysis

Consumer liking data were analyzed by multivariate analysis of variance (MANOVA). When a significant difference (*p* < 0.05) was detected, a *t*-test with Holm correction was applied to evaluate the difference between the samples. Pearson coefficients were calculated to evaluate the correlations between the consumer liking scores gained in different evaluation conditions. Principal component analysis (PCA) was performed on the individual consumer scores in three different conditions using the correlation matrix. PCA models were validated using the full cross-validation procedure. The correspondence analysis (CA) was performed on the frequency table containing responses to the check-all-that-apply questions. Statistical analyses were performed using the R-language and XLSTAT software (Addinsoft, Paris, France) packages.

## 3. Results

### 3.1. Consumer Liking of Apple Juices in Different Evaluation Conditions

The same consumer group evaluated the quality of eight apple juices in three testing conditions in our laboratory study. The results of consumer evaluations expressed on the 9-point hedonic scale are presented in Table 2.

The results of the consumer study in blind condition were described in detail and discussed in a previous paper, as regards physicochemical and sensory characteristics of juices [28]. Presently, we used these results to compare them with the consumer ratings in expected and informed conditions. The consumers reacted differently to the sensory characteristics of the juices. The overall liking scores in blind conditions ranged from 4.1 corresponding to dislike slightly to 6.2 corresponding to like slightly. Significant differences were found in consumer liking of the samples assessed in a blind test (*p* < 0.05). Freshly squeezed juices (samples G and H) and reconstituted cloudy juices (samples C and D) were the most liked juices, while cloudy NFC juices (samples E and F) and reconstituted clear juices were the most disliked. The results are in line with those reported earlier [31], where average consumer ratings of the studied apple juice were at 5.5–6.0 on a 9-point scale, and these rather low ratings were explained by consumer habits for certain products.

The expected liking scores were in the range of 5.8–7.1 and higher than blind scores. These results indicate that the package with its product information, including brand, type of production, nutritional value, etc., generates high consumer expectations. Significant differences were observed in mean consumer scores; fresh juices (G, H) and cloudy FC juice (D) gained the highest ratings, while clear juice (A) and naturally cloudy (E) the lowest. The NFC juice (E) which is an organic product (information on the package) received the lowest rating among the studied juices, as it had a large amount of natural sediment. Note that sediment and turbidity of fruit juice may be perceived negatively by consumers as a product defect [32]. On the other hand, consumers were reported to have higher expectations for local apple juices as opposed to the mainstream juices available on the market, and also expected the higher quality of fresh apple juices as opposed to juices subject to thermal, high hydrostatic pressure, or pulsed-electric field treatment [21,22].

Our present study explored consumer perceptions of apple juices based on the comprehensive product information available on the label. The consumers, knowing the product brand and product information available on the package, rated the sensory quality of fresh juices (G, H) the highest. Significantly lower scores were obtained by clear juices (A, B) and naturally cloudy NFC (E, F) juices. Similar results were reported by Lee, Lusk, Mirosa, and Oey [22], indicating that consumers mostly valued the untreated apple juices.

When comparing consumer liking of juices in various testing conditions, the highest influence of product information on liking was observed for NFC juices (E, F). These samples correspond to the premium brands available in glass bottles. Clear FC juices (A, B) and fresh ones (G, H) were also assessed higher in informed as compared to blind tests. The blind and informed liking did not differ significantly only for cloudy FC juices (C, D). These latter juices were available in cartons and received high ratings for sensory quality at the first stage of the study. On the other hand, the expected quality scores were higher than the sensory scores for coded samples for six of the eight juices tested (except samples C and G, which received high sensory evaluation). The expected liking scores were significantly higher than the informed liking scores for clear (A, B) and naturally cloudy NFC (E, F) juices. The discrepancy between these assessments indicates that the sensory profiles of these juices do not meet consumer expectations generated by the product information contained on the packaging.

**Figure 1:**
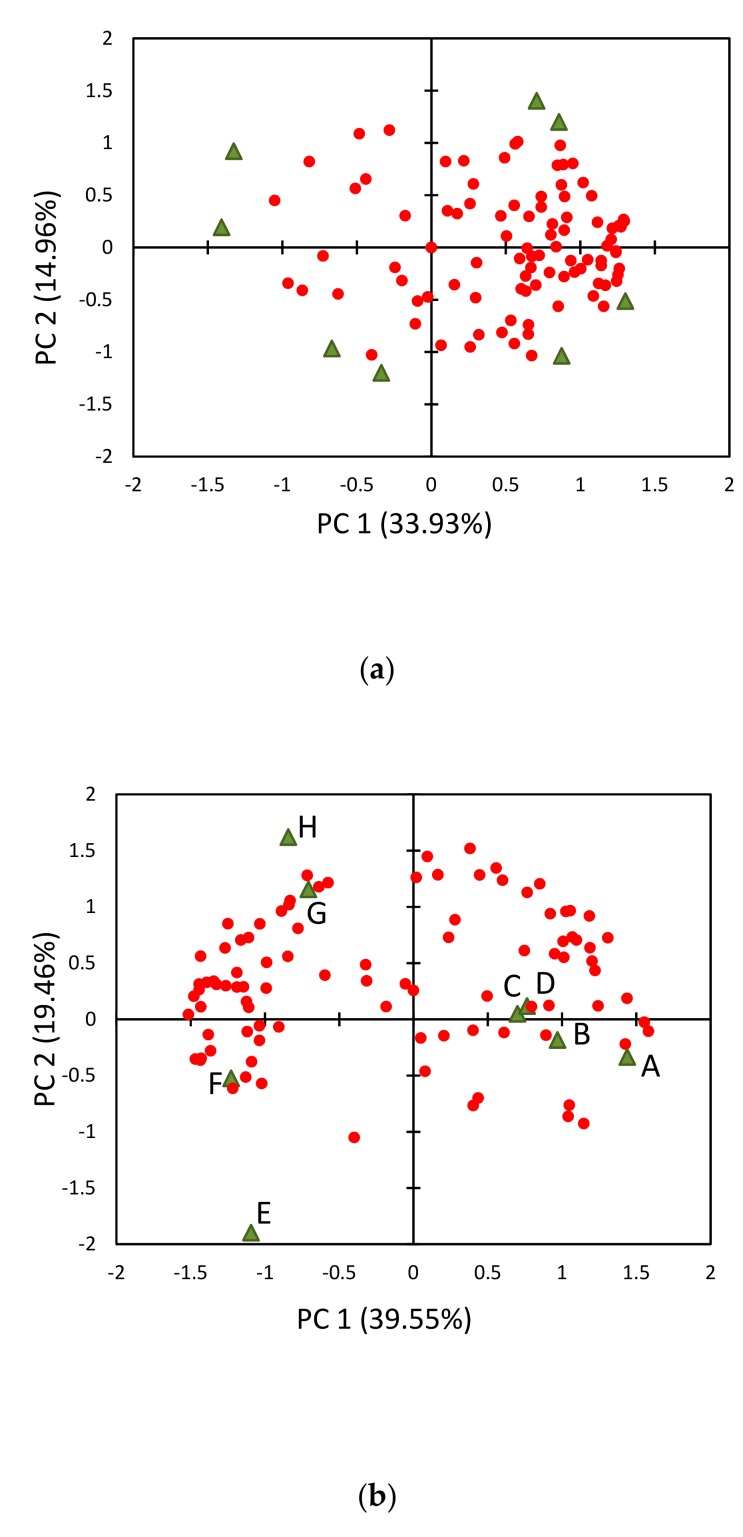

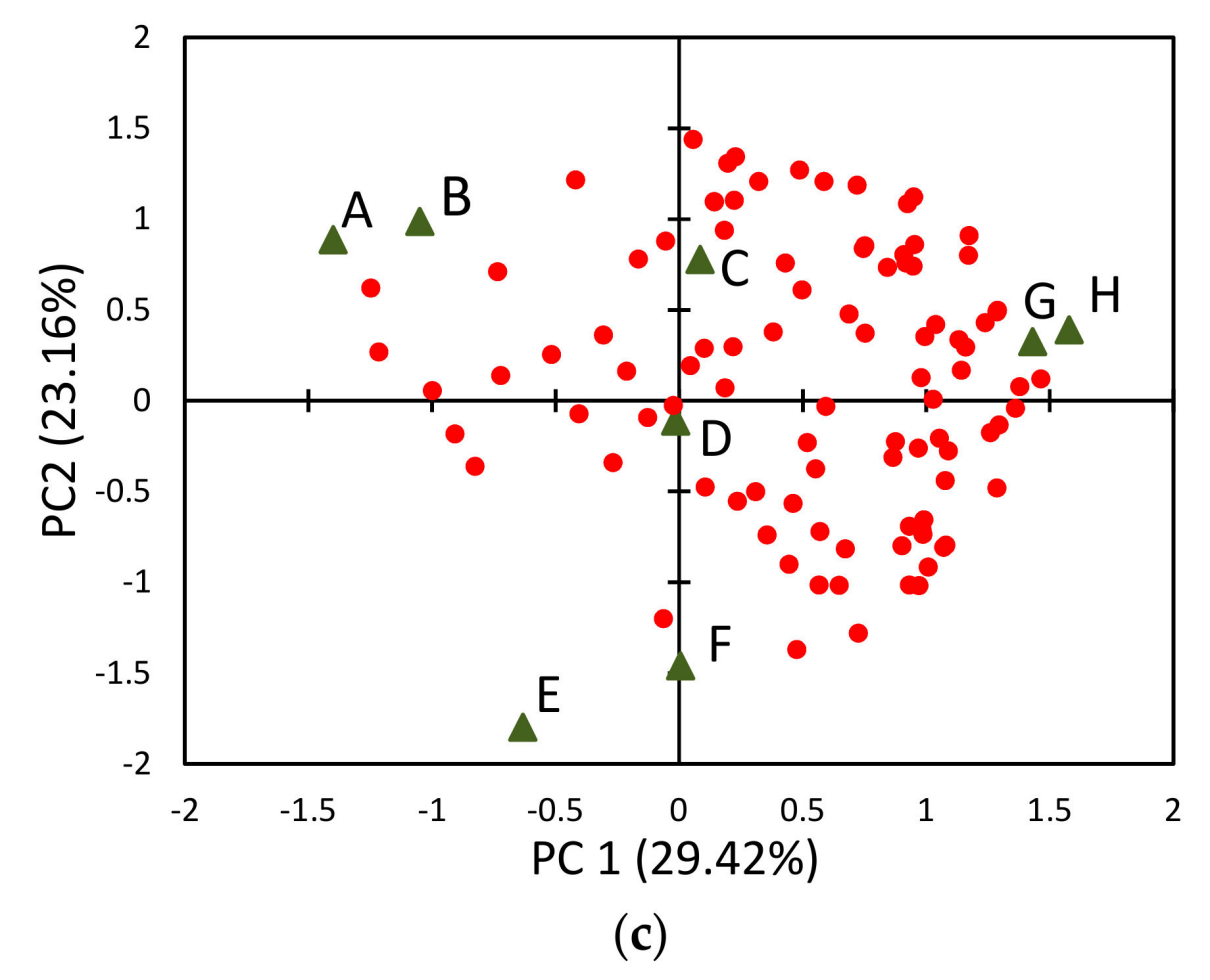
Internal preference maps for different testing conditions: (**a**) blind, (**b**) expected, (**c**) informed. A,B—clear FC juices; C,D—cloudy FC juices; E,F—cloudy NFC juices; G,H—fresh juices. Red dots correspond to the individual consumers. PC1 and PC2 correspond to the first and second principal component, respectively.

The mean consumer liking scores are useful for determining the overall trends, but do not provide information on the formation of the consumer groups (segments) with similar preferences [33]. Consumer liking data were analyzed using principal component analysis (PCA), performed on the individual consumer scores obtained in three different conditions. Internal preference maps were generated, illustrating the main preference directions [34], presented as biplots in Figure 1.

The distribution of individual consumer scores varied depending on the testing conditions. Regardless of the testing conditions, the two juices in each of the pairs belonging to a certain product category were rated similarly by consumers; thus, the points corresponding to these juices are located close together in the two-dimensional space.

Most of the consumers preferred cloudy FC and freshly squeezed juices in the blind test (Figure 1a). The first PC1 component distinctly differentiated the juices according to sensory preferences (direct vs. reconstituted juices). A very different distribution of consumers and juices was obtained for expected liking (Figure 1b), where two main groups of the consumers may be distinguished. The first group assigned higher ratings to NFC juices, available in glass bottles. The second group, more scattered, rated FC juices, clear and cloudy varieties, available in 1 L boxes higher. As shown in Figure 1c, combined internal and external attributes provided poorer differentiation of the juices studied. Note that PC1 clearly differentiated clear FC juices from fresh juices, but not pasteurized cloudy FC and NFC juices. Most consumers, basing on sensory quality and external attributes, assessed the quality of fresh juices (G, H) as higher. The preference map for informed testing differed significantly from that for blind testing. These results confirmed the strong influence of the external attributes on the sensory evaluation of apple juice. Similar results were obtained by Lee, Lusk, Mirosa, and Oey [22].

There was a strong positive correlation (*p* < 0.05) between the expected and informed liking scores (*r* = 0.944) and between the blind and informed consumer scores (*r* = 0.800). No correlation was observed between blind and expected liking, indicating that the two approaches measure different aspects of product quality. These results are in agreement with the findings reported by Varela, Ares, Giménez, and Gámbaro [35].

### 3.2. Perception of Apple Juices Based on External Attributes 

In order to gather supplementary data on the consumer perception of the juices studied, a check-all-that-apply (CATA) questionnaire with terms related to characteristics of the product and the brand image was used. CATA questions are increasingly more often used in consumer research to investigate the perceptions of a variety of attributes perceived by consumers and to obtain a rapid product profile from consumers [36,37,38].

Consumers used between one and seven terms to describe the apple juices in both laboratory and online studies. The most frequently used terms were natural and tasty; the least used term was tasteless. Most consumers indicated both cloudy NFC (E, F) and fresh (G, H) juices as natural. These juices were rated also as very healthy and expensive. The differences between FC and NFC juices in their naturalness were clearer in the laboratory as compared to the online study. This may be due to increased attention of the respondents and the lack of a time limit in the laboratory testing. Furthermore, consumers perceived the juices studied as more innovative and trendier in the classic laboratory study as compared to the online experiment. This may be due to the fact that the photos did not fully reflect the differences between the products. Based on the results obtained, we concluded that apple juices are generally perceived by the consumers as natural and healthy products.

**Figure 2:**
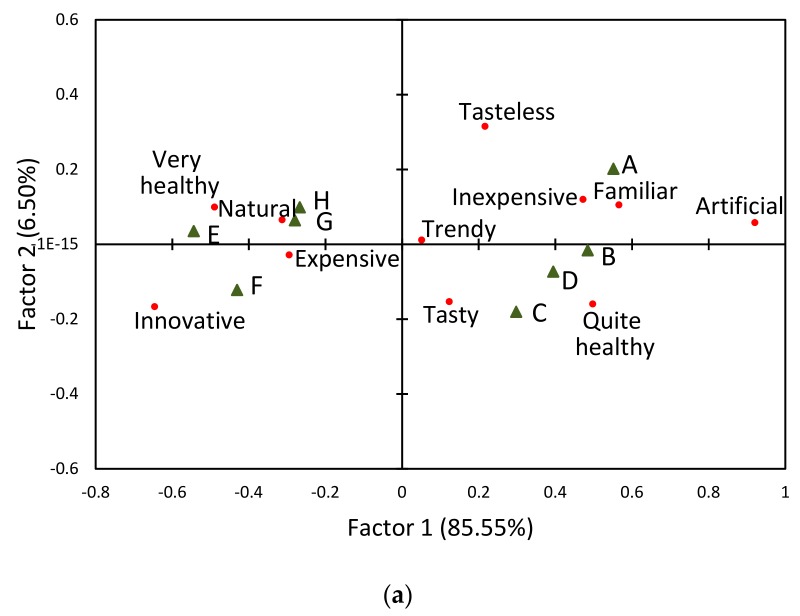

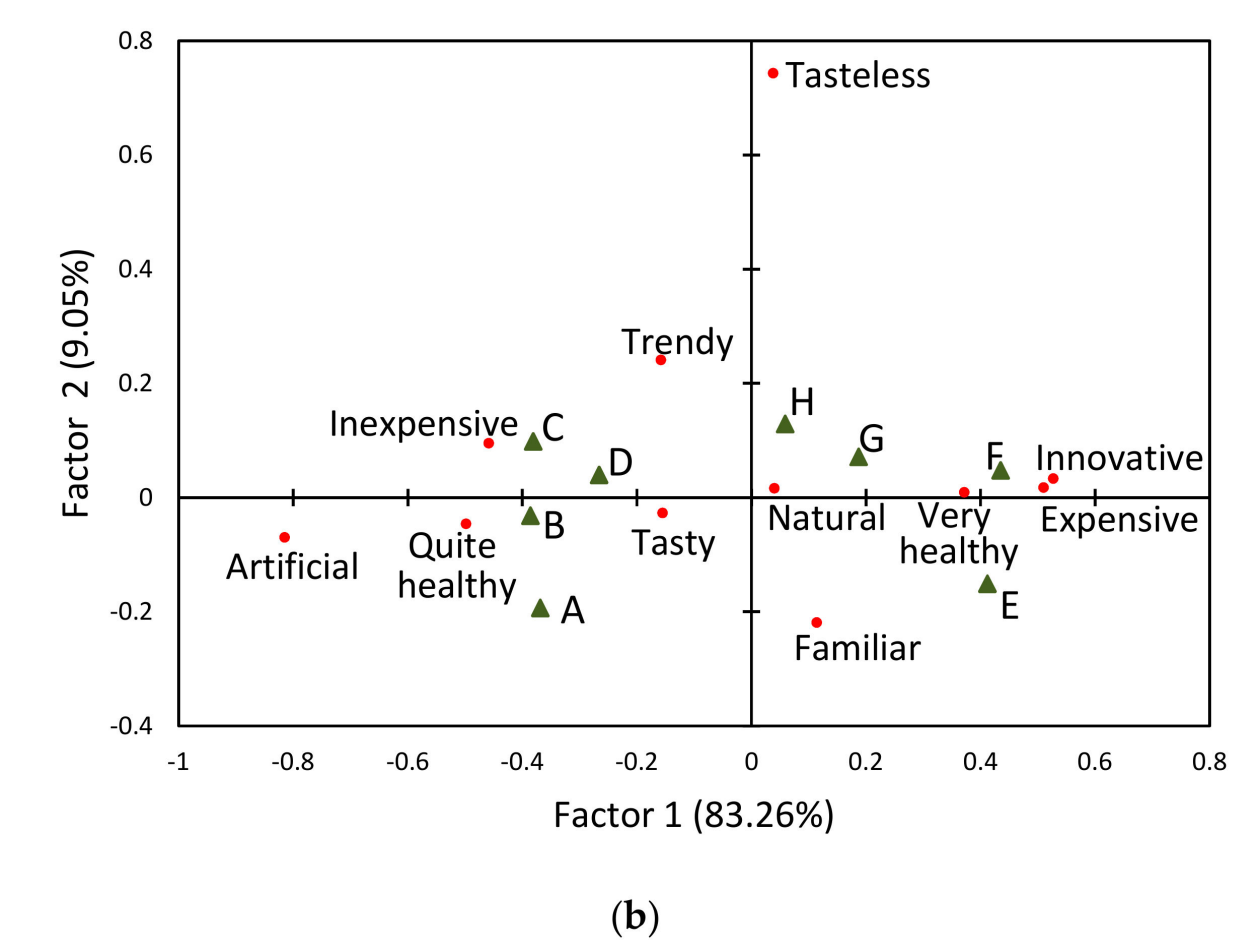
Correspondence analysis of check-all-that-apply responses: (**a**) laboratory consumer study, (**b**) online consumer study. A,B—clear FC juices; C,D—cloudy FC juices; E,F—cloudy NFC juices; G,H—fresh juices.

The correspondence analysis was used to visualize and interpret the results of the check-all-that-apply questions [39]. The plots of the first two factors for the consumer laboratory study and the online study data are shown in Figure 2.

A two-dimensional representation of the data was obtained, describing respectively 92.05% and 92.31% of the variability of laboratory and online consumer data. The results of the correspondence analysis showed that the external attributes differentiated the studied juices. The first direction differentiating the products was related to the perceived naturalness. The second dimension was related to the expected taste of the juice and was positively correlated to tasteless and negatively to tasty. As shown in Figure 2, the first and the second dimensions sorted the samples into the two main groups, according to consumer expectations: the first group contained FC juices (A, B, C, D) and the second group direct juices (E, F, G, H). The sample distribution was distinctly different from that obtained for blind and informed liking scores, while similar to the distribution of expected liking scores (Figure 2). As a result of both experiments, FC juices were perceived as more artificial, quite healthy, and inexpensive, whereas NFC juices were perceived as more healthy, natural, innovative, and expensive. These characteristics may be explained by the type of packaging and the production information declared by the manufacturer on the package. Note that NFC juices are available in glass bottles, which makes the natural sediment more visible.

CATA results obtained in the laboratory and online studies were convergent. This suggests that an online experiment could be an effective alternative to the classic consumer laboratory study, being a faster and cheaper way of obtaining consumer perception data.

### 3.3. Visual Attention to Product Labels

To examine the labels’ zones that attract consumer attention, an online study was conducted using the innovative Attensee.com application. Based on the defined reference areas (RA), the application generated three types of data which are similar to the data obtained by the eye-tracking tool: (1) path of engagement specifying locations on packaging which attracted the attention of the respondents; (2) heatmap for time, showing the average time that respondents focused on an area; and (3) heatmap for attention, specifying the number of people who focused on a particular area of an image.

Firstly, the image of all the analyzed products was presented to consumers. The purpose of this presentation was to investigate which products attract the most consumer attention during the decision process on the store shelf.

During the on-shelf product recognition survey, consumers mostly focused on NFC juices (samples E and F) from local producers and focused the least on FC juices from well-known brands. According to the CATA questionnaire results discussed above (Section 3.2), it can be presumed that customers were focusing longer on products that they considered more attractive (healthy, natural, innovative) and from the premium segment (expensive). Consumer interest could also be due to the fact that NFC juices produced by local producers are less recognized than FC juices from well-known brands available nationwide. This feature may be seen by consumers as positive. Hempel and Hamm [40] examined consumer preferences for different food products of varying places of origin and observed that consumers prefer locally produced food.

Based on our results, we may distinguish the packaging elements that attract consumer attention. First of all, consumers paid attention to brand names of products, but it should be emphasized that they focused more attention on local juices as opposed to well-known brands. Subsequently, consumers noticed the information about product type and quality. More specifically, over 99% of consumers noticed the green BIO-label on NFC juice E. Studies show that consumers have a high preference for ethical or ‘green’ products and favor environmentally labeled packaging as the most important criteria in food choice [41,42]. Furthermore, consumers focused for over 2 s on average on the area of NFC juice F informing about type of product (“pressed apple juice”). 

One more experimental survey concerned the individual label pictures of juices from different product categories. Table 3 presents the aggregated results of this study. 

Generally, the longest average attention time (1.2 s) was devoted to the part of labels with nutritional information and involved almost 94% of consumers. Based on a detailed analysis of the consumer results and reference areas for the individual products, we noticed that the more clearly designed and sharply outlined nutritional information caused shorter attention time to this part of the label.

The brand names of juices were explored by almost 90% of the consumers who focused on this part of the label for an average of 0.5 s. Analyzing the percentage of consumers who focused their attention on the label’s specified reference area, we found that the products from the better-known brands (clear and cloudy FC juices) attracted less attention than little-known craft brands (freshly squeezed and NFC juices). 

The third main area of the consumer interest was the part of the label describing the type of product. About 87% of consumers focused their attention on that area for 0.8 s on average. In this case, the differences in the time of gaze fixation for all of the studied products was not significant and fluctuated between 0.7 and 1.2 s. 

The next two areas, also important from the consumer point of view, were the information about the manufacturer/producer and places with special pictograms and signs including certificates and pictograms describing social action. The interest in manufacturer information was visible especially in the case of the NFC juices (samples F and E)—88% and 78% of consumers visited these areas and spent about 0.5 and 0.9 s, respectively, focusing on it. Special signs about sports and social action available on the package of juice D attracted the attention of 59% consumers, who spent about 0.7 s focusing on it. Furthermore, the culinary certificate on the label of juice F was noticed by 59% consumers who focused their gaze on it for 0.7 s on average.

When analyzing the heatmaps of a single juice label, we noted that consumers did not study some parts of the product information. This suggests that consumers did not assess the presented information comprehensively. These observations are in agreement with findings published by Oliveira et al. [15]. Generally, consumers mainly turn their attention to the information given in larger letters, information presented in an intense color (especially red), and special pictograms like certificates. It should be emphasized that we presented the labels to the consumers on a computer screen one by one. The consumers had possibility to also investigate the information presented on the sides and back of the packages. The consumer attention to product information in real-life conditions is expected to be even lower than presently reported.

It was previously reported that regardless of the type of product and the label design, consumers direct their attention to selected label zones such as brand, ingredients, and nutritional information [15,43]. The results obtained in the present study for real market products are convergent with those reports. The proposed approach would be useful for assessing the effects of a variety of commercial juice packaging designs on consumer perception.

## 4. Conclusions

The present study combined sensory evaluation and visual attention experiments to investigate how intrinsic and extrinsic factors affected consumers’ perceptions of apple juice quality. Eight commercial apple juices were studied, representing the four product categories currently available on the Polish market, which were evaluated by consumers. In a blind test, consumers rated the experienced quality of fresh and cloudy FC juices as the highest and the quality of cloudy NFC juices as the lowest. The packages with the product description not only generated high consumer expectations, but also impacted the sensory liking in a markedly positive way. For most of the tested juices, consumers rated the expected quality higher than the experienced quality of the coded samples. The product information influenced the rating of the quality of NFC juices, fresh juices, and clear FC juices in a significant and positive manner. These results confirmed the importance of the information presented on the packaging. On the other hand, they indicated the need to improve the sensory quality of juices to meet consumer expectations and encourage them to buy the product again.

An innovative online visual attention tracking technique was used for a more detailed analysis of the perception of packaging and labels. The online shelf test revealed that consumers focused the most on NFC juices from local producers. Additional information about the perceived juice quality in both the laboratory and online studies was provided by CATA questionnaires. The results obtained in all experiments were convergent and revealed that consumers perceived NFC juices as more natural, healthy, and expensive than juices reconstituted from concentrate.

The study of individual packaging enabled to identify zones that attracted consumers’ visual attention. The consumers predominantly focused on nutritional data, brand name, and information about the type of product. This knowledge may be of practical utility for packaging and label design, to improve the attractiveness and competitiveness of the product. However, the observed effects might be modified by many factors including participants’ previous knowledge of the product, text information presented with the image, and the level of congruency of the images. These aspects will be explored more fully in future research.

We conclude that the experimental approach proposed in this study provided a comprehensive view of the influence of the product information presented on packaging on the consumer expectations and sensory perception; moreover, it enabled to identify the important features of the packaging and labels. The results confirmed the effect of intrinsic and extrinsic factors on the product quality perception.

## Figures and Tables

**Table 1:** Description of the apple juices used in the study.

Juice ^1^	Product Category ^2^	Package	Market Segment
A	FC, clear	1.00 L box	Standard
B	FC, clear	1.00 L light plastic bottle	Standard
C	FC, cloudy	1.00 L box	Standard
D	FC, cloudy	0.70 L light glass bottle	Standard
E	NFC, cloudy, pasteurized	0.25 L dark glass bottle	Premium
F	NFC, cloudy, pasteurized	0.25 L light glass bottle	Premium
G	NFC, freshly squeezed	0.25 L light glass bottle	Premium
H	NFC, freshly squeezed	0.25 L light glass bottle	Premium

^1^ The set of A–H juices was the same as studied previously [28]. ^2^ FC—from concentrate, NFC—not from concentrate.

**Table 2:** Mean consumer liking scores^1^ of apple juices evaluated in blind, expected, and informed conditions.

Juice	Evaluation Conditions ^1^		
	Blind ^2^	Expected	Informed
A	4.5 ^c,d,C^	5.8 ^c,A^	5.1 ^e,B^
B	4.9 ^b,c,C^	6.1 ^b,c,A^	5.4 ^d,eB^
C	5.8 ^a,A^	6.4 ^a,b,c,A^	6.3 ^b,c,A^
D	5.6 ^a,bB^	6.6 ^a,b,A^	6.1 ^b,c,d,A,B^
E	4.1 ^d,C^	5.8 ^c,A^	5.3 ^d,e,B^
F	4.2 ^c,d,C^	6.4 ^b,c,A^	5.7 ^c,d,e,B^
G	6.2 ^a,B^	6.7 ^a,b,A,B^	6.9 ^a,b,A^
H	5.7 ^a,B^	7.1 ^a,A^	7.3 ^a,A^

^1^ Evaluated on a nine-point hedonic scale. ^2^ The consumer ratings in blind conditions were available from our previous study [28]. Different lowercase superscripts (a–e) within a column indicate significant differences according to the *t*-test (*p* < 0.05). Different capital superscripts (A–C) within a row indicate significant differences according to the *t*-test (*p* < 0.05).

**Table 3:** Average percentage of consumers who focused their attention on the label’s specified reference area defined individually for each type of label, and the average time of attention for apple juices assessed using a visual attention tracking tool.

Juice	Average Percentage of Consumers (%)			Average Time of Attention (s)		
	Brand	Nutritional Label	Type of Product	Brand	Nutritional Label	Type of Product
A	87	94	74	0.3	2.2	1.2
B	87	100	100	0.6	0.8	0.8
C	85	97	70	0.6	2.1	0.7
D	76	84	84	0.5	0.9	1.0
E	93	83	91	0.7	0.4	0.7
F	95	97	86	0.2	1.2	0.7
G	99	100	100	0.4	0.3	1.0
H	95	95	87	0.4	1.4	0.7
Mean	89.6	93.8	86.5	0.5	1.2	0.8
